# Robustness of model-based high-resolution prediction of forest biomass against different field plot designs

**DOI:** 10.1186/s13021-015-0038-1

**Published:** 2015-12-02

**Authors:** Virpi Junttila, Basanta Gautam, Bhaskar Singh Karky, Almasi Maguya, Katri Tegel, Tuomo Kauranne, Katja Gunia, Jarno Hämäläinen, Petri Latva-Käyrä, Ekaterina Nikolaeva, Jussi Peuhkurinen

**Affiliations:** 1 Department of Mathematics and Physics, Lappeenranta University of Technology, P.O.Box 20, 53851 Lappeenranta, Finland; 2 Arbonaut Ltd, Kaislakatu 2, 80130 Joensuu, Finland; 3 International Centre for Integrated Mountain Development (ICIMOD), G.P.O. Box 3226, Khumaltar, Lalitpur, Kathmandu, Nepal; 4 Mzumbe University, P.O. Box 1, Mzumbe, Morogoro, Tanzania

**Keywords:** Above-ground biomass, LiDAR, REDD+, Participatory forest monitoring

## Abstract

**Background:**

Participatory forest monitoring has been promoted as a means to engage local forest-dependent communities in concrete climate mitigation activities as it brings a sense of ownership to the communities and hence increases the likelihood of success of forest preservation measures. However, sceptics of this approach argue that local community forest members will not easily attain the level of technical proficiency that accurate monitoring needs. Thus it is interesting to establish if local communities can attain such a level of technical proficiency. This paper addresses this issue by assessing the robustness of biomass estimation models based on air-borne laser data using models calibrated with two different field sample designs namely, field data gathered by professional forester teams and field data collected by local communities trained by professional foresters in two study sites in Nepal. The aim is to find if the two field sample data sets can give similar results (LiDAR models) and whether the data can be combined and used together in estimating biomass.

**Results:**

Results show that even though the sampling designs and principles of both field campaigns were different, they produced equivalent regression models based on LiDAR data. This was successful in one of the sites (Gorkha). At the other site (Chitwan), however, major discrepancies remained in model-based estimates that used different field sample data sets. This discrepancy can be attributed to the complex terrain and dense forest in the site which makes it difficult to obtain an accurate digital elevation model (DTM) from LiDAR data, and neither set of data produced satisfactory results.

**Conclusions:**

Field sample data produced by professional foresters and field sample data produced by professionally trained communities can be used together without affecting prediction performance provided that the correlation between LiDAR predictors and biomass estimates is good enough.

## Background

Greenhouse gas (GHG) emissions from tropical deforestation and forest degradation contribute about 15–20 % of total annual global GHG emissions, making them the second largest source of greenhouse gases globally [1]. To reduce especially CO_2_ (carbon dioxide) emissions from the forestry sector, the United Nations has established a program that would provide payments for the reduction of emissions from deforestation and forest degradation (REDD+). REDD+ is a performance-based policy instrument aimed at reducing anthropogenic emissions of GHG [2, 3].

Nepal is one of the countries participating in REDD+. After successful implementation of a Community Forestry programme, Nepal has taken another leap by piloting innovative REDD+ projects. The International Centre for Integrated Mountain Development (ICIMOD) is one of the first organizations to implement a community-based REDD+ pilot at micro-watershed level. ICIMOD and its partners, the Federation of Community Forestry Users, Nepal (FECOFUN) and the Asia Network for Sustainable Agriculture and Bioresources (ANSAB) implemented a pilot project from 2009–2013, with support from the Norwegian Agency for Development Cooperation (NORAD) climate and forest Initiative [4]. The major focus of the project was to develop and demonstrate an innovative benefit-sharing mechanism for REDD+ incentives using institutionally and socially inclusive approaches to address the drivers of deforestation and forest degradation and improve forest governance [5] in three micro-watersheds, namely Kayarkhola in Chitwan, Ludikhola in Gorkha and Charanawati in Dolakha districts. The pilot project focused on sequestering carbon through community-based forest management. It is one of the first carbon offset demonstration projects in the world that involves local communities in monitoring the carbon in their forests and providing the necessary training for them to do so. Training on assessing forest carbon pools was provided to the local communities that manage the forest [6]. The trained local communities collected field plot data from 2010 to 2012. The results of this effort are summarized in [7].

In a joint effort, the Forest Resource Assessment Nepal project, Arbonaut Ltd. and ICIMOD carried out a wall-to-wall airborne discrete-return light detection and ranging (LiDAR) data and subsequent field plot collection in two watersheds in Chitwan and Gorkha. The main aim of the work was to estimate accurately and with a high spatial resolution the forest above ground biomass (AGB)/carbon in the watersheds. The field data was collected by professional foresters and technicians.

In measuring biomass for calculating REDD+ compensation the measurements should be conducted in a biennial manner, as has been recently agreed at a UNFCCC meeting in Bonn [8]. As national measurements are required at such a high frequency, traditional sampling-based methods become prohibitively expensive. We therefore propose to use a model-based strategy for biomass prediction, where field plots are only used for model calibration, i.e. model parameter estimation, and biomass prediction models are based on remotely sensed data, such as LiDAR, which is used in this study, or satellite imagery.

In this study, plot-level AGB values estimated from field measurements (labelled as “AGB field estimates” in this article) in given inventory areas are predicted (“predictions”) in new validation plot locations (“validation set”) using linear model which is calibrated using field measurements obtained from the respective areas (“training set”). The field measurements are collected by two types of forest inventory teams: a professional measurement team (“Prof”) and measurement teams trained from the local community members (“Comm”). The local community members were trained to do forest inventory work by professionals from ICIMOD and its project partners that operated independently of the professional measurement team. Inventory work, i.e., field sample plot selection and actual measurement work, is thus performed by two separate groups, which may lead to data sets with different characteristics, even when the measurements are conducted in the same area.

To justify the use of participatory forest monitoring and inventory work of local communities, it is important that there are no significant differences in AGB predictions based on field measurements of both teams. Thus, it is needed to verify if different data sets from the same area lead to similar model based predictions, and if the datasets can be combined and used together in predicting AGB for the areas in the future. In this study, we first analyse the field estimates measured by both teams and validate the correlation between the model auxiliary data, LiDAR predictors, and the corresponding field estimates. We then validate the robustness of high-resolution model-based biomass estimation against different sampling designs from the same area. Models using different sources of training set data but resulting in the same predictions with the same validation data are considered robust against the training set source.

## Results

**Fig. 1:**
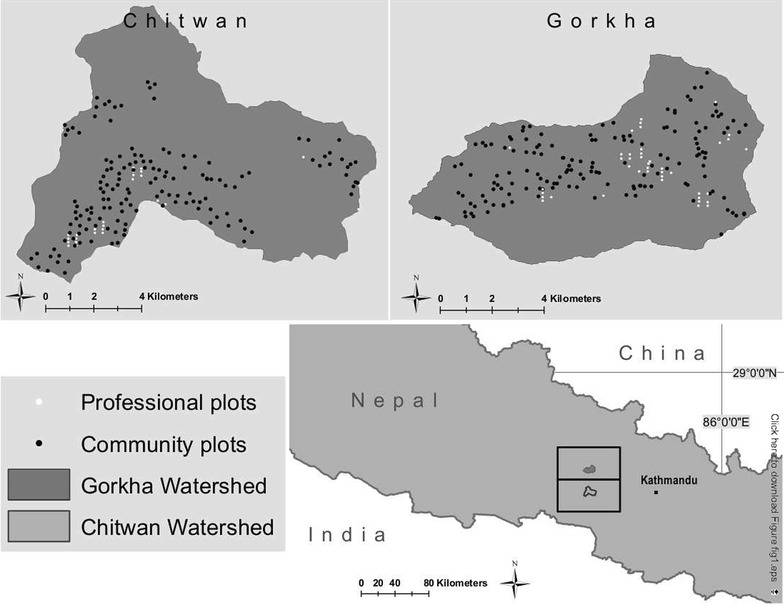
Maps of the study area showing the two watersheds in Chitwan and Gorkha. The map on *top* shows the ICIMOD plots used in this study

**Fig. 2:**
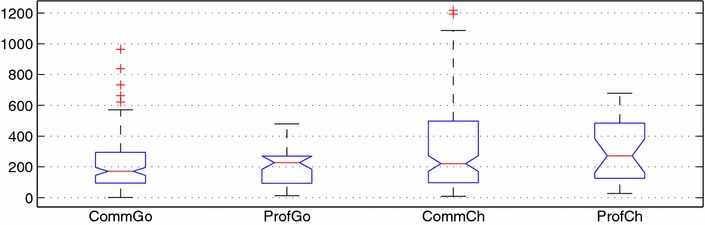
Boxplots of AGB field estimates in plots located in closed type community owned forests. Median, 25th and 75th percentiles and outliers are shown

The model-based predictions and corresponding error analyses were performed using data collected from two inventory study sites located in Nepal, namely Gorkha (labelled as “Go”) and Chitwan (“Ch”), see Fig. 1 for the location of the sites. The scope in forest inventory by the different inventory teams was different. The professional team measured only closed canopy forest owned by both the state and by the forest communities. The local field teams measured both open and closed canopy forests but only those owned by communities. Only the field plots of similar forest types, plots from closed canopy forest owned by communities, were used in this study. The number of plots measured by the community teams located in closed type forests that are owned by the communities is 151 in study site Gorkha and 151 in study site Chitwan, while the number of plots measured by the professional teams located in closed type forests owned by the communities is 41 in Gorkha and 26 in Chitwan. See Fig. 1 for plot locations and Fig. 2 for the distribution of field estimates.

**Fig. 3:**
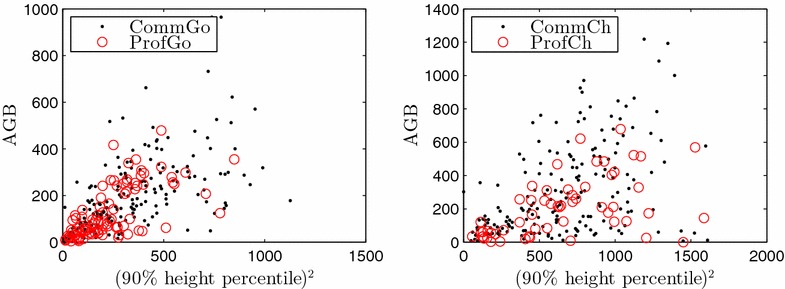
An example of LiDAR predictor—AGB field estimate scatterogram

Examples of the combined distributions of LiDAR predictor values and AGB field estimates of each sample are shown in Fig. 3. The LiDAR predictor—AGB field estimate correlation of both sample in Gorkha, the plots measured by professional teams and the plots measured by the members of local communities, is higher than in Chitwan. The correlation of AGB and the LiDAR example predictor in Gorkha is 0.66 for sample ProfGo, 0.59 for CommGo, and in Chitwan it is 0.52 for ProfCh and 0.45 for CommCh. With visual analysis, the LiDAR predictor—AGB distributions of different inventor teams are similar in both study sites. Similar properties hold for each predictor used in this study.

### Results of study site Gorkha

**Fig. 4:**
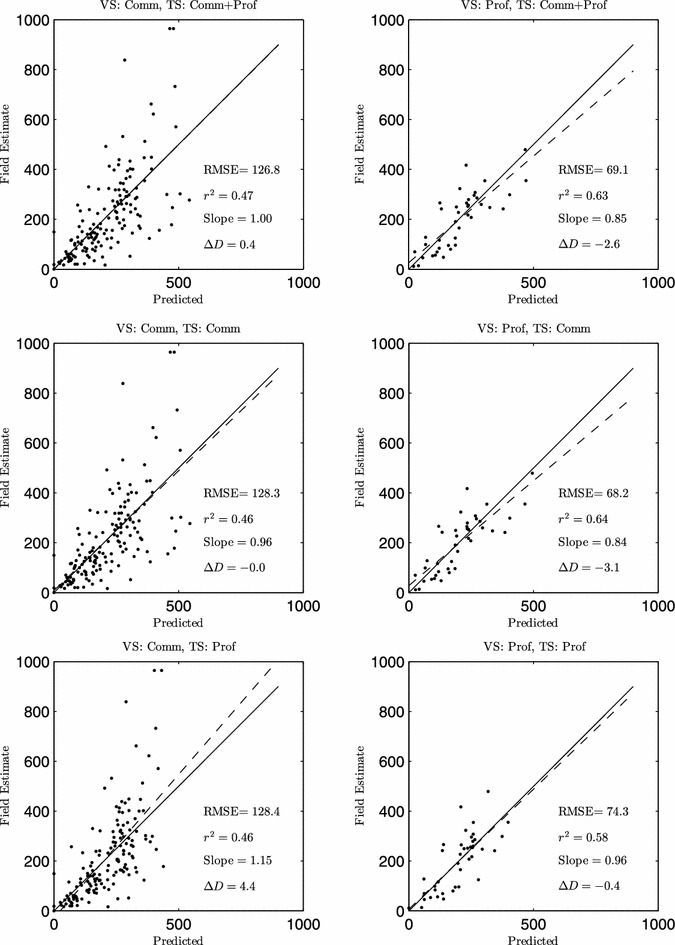
Scatterogram and prediction error analysis of AGB predictions and field estimates in study site Gorkha. *VS* validation set, *TS* training set

**Fig. 5:**
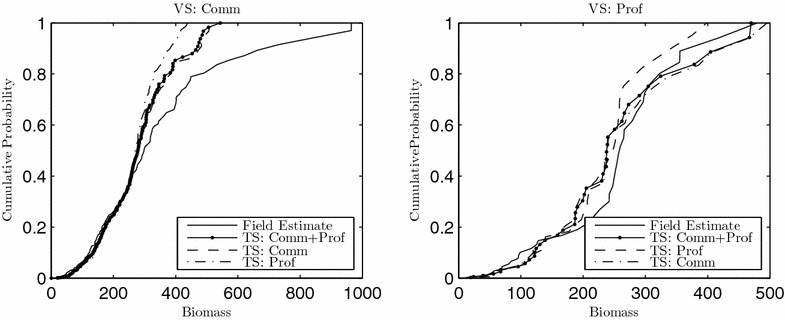
Cumulative distribution of plot-level AGB field estimates and plot-level AGB predictions estimated with models based on different training subsets in study site Gorkha

The results for predictions in study site Gorkha are shown in Table 1 and Figs. 4 and 5. In the table, model prediction precision and accuracy are evaluated by relative root mean square error (RMSE %) and relative mean difference (D %). The prediction error distributions in two validation sets (Comm and Prof) resulting from three different model training set data (Comm, Prof and the combined set of these plots, Comm + Prof) are verified. The differences of the mean values (D %) between the self-validation (validation set and training set are the same) and the cross-validation cases (training set is different than validation set) are tested by a two-sample *t* test with a 5 % significance level against the case where the validation set and training set are the same. Similarly, the difference of the RMSE % values are tested with a two-sample *F* test for equal variances with a 5 % significance level. The tests show that distributions of prediction error among different models in the validation set Comm are similar. The mean of the errors are the same as that for training set Comm (t test p values $>0.79$ for training sets Prof and Comm + Prof) and also the variance of the error distributions are similar (variance test p values $>0.89$). Similar results are shown for the AGB predictions in the plots measured by the professional teams. The statistical tests for the prediction error show no significant difference in error mean or error variance compared to the predictions estimated with training set Prof (t test p values are $>0.84$ and variance test p values are $>0.59$ for training sets Comm and Comm + Prof). Figure 4 shows that independent of the training and validation set used, a relatively good model fit is obtained for validation set Comm ($r^2 \ge 0.46$ for all training sets) and for validation set Prof ($r^2\ge 0.58$ for all training sets).

**Table 1:** Results with different combinations of training set–validation set in study site Gorkha

Training set	Error stat.		Test against baseline, p values	
	RMSE %	BIAS %	Variance test	t test for mean
Validation set: Comm				
Comm (baseline)	59.2	0.0		
Prof	59.3	−1.8	0.994	0.794
Comm + Prof	58.6	−0.2	0.888	0.977
Validation set: Prof				
Prof (baseline)	37.5	0.2		
Comm	34.4	1.9	0.586	0.839
Comm + Prof	34.8	1.5	0.644	0.872

Also the distributions of the AGB predictions shown in Fig. 5 are close to each other. The lack of ability to predict accurately the largest AGB values in plots measured by the Community teams is similar when using any of the training sets (see the left sub-figure). Up to about 300 Mg/ha, the AGB distributions of all predictions are very close to the distribution AGB field estimates of Community teams, the larger AGB values tend to be under-estimated. In case of the AGB field estimates of the Professional teams, predictions estimated by different training sets of either of the teams result in quite correct AGB distributions. No big deviations can be seen up to 400–500 Mg/ha.

### Results of study site Chitwan

**Fig. 6:**
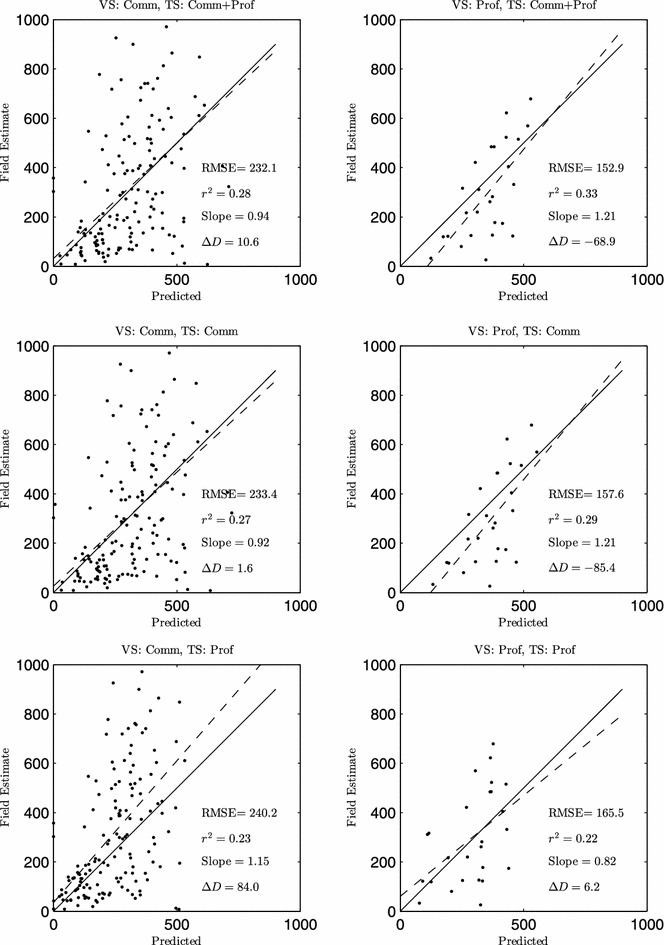
Scatterogram and prediction error analysis of the AGB predictions (“predicted”) and AGB field estimates in study site Chitwan

**Fig. 7:**
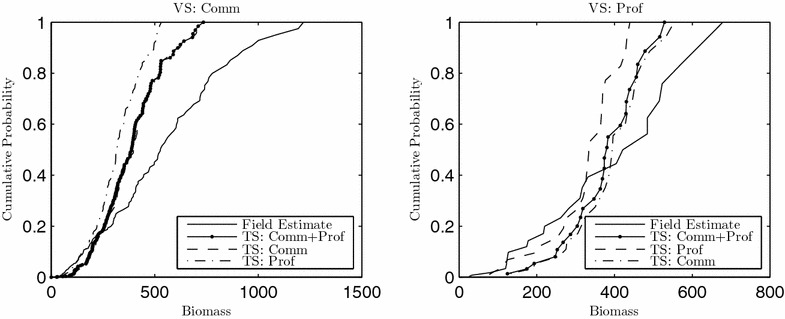
Cumulative distribution of plot-level AGB field estimates and plot-level AGB predictions estimated with models based on different training subsets in study site Chitwan

Results of AGB predictions in study site Chitwan are shown in Table 2 and Figs. 6 and 7. The variance test in Table 2 shows no significant difference between the prediction precision (RMSE %) between the predictions obtained with different training sets for either validation sets (*p* values $>0.81$ for validations set Comm, $>0.43$ for Prof). However, the t test for the average values of the average relative differences, D %, shows significant difference (*p* value $0.008<0.05$) for prediction of community team plots using model calibrated with field estimates of professional teams. Similarly, predictions for professional team measured plots predicted with model calibrated using the community team plots, the *p* value is close to 0.05 although not less than it. Thus, there is significant average error in predictions when the training set and validation set are different. Similar doesn’t hold when the training set consists also data from the validation set (Training set Comm + Prof).

**Table 2:** Results with different combinations of training set–validation set in study site Chitwan

Training set	Error stat.		Test against baseline, pvalues	
	RMSE %	BIAS %	Variance test	t test for mean
Validation set: Comm				
Comm (baseline)	72.1	−0.5		
Prof	74.2	−22.6	0.806	0.008
Comm + Prof	71.7	−3.5	0.931	0.720
Validation set: Prof				
Prof (baseline)	55.5	−2.5		
Comm	52.9	23.6	0.433	0.079
Comm + Prof	51.3	19.0	0.452	0.146

The one-to-one scatterograms in Fig. 6 shows that the correlation between AGB field estimates and model based predictions is poor in each cross-validation set. Even the predictions estimated using the same training set and validation set (self-validation: validation set Comm estimated with training set Comm and validation set Prof estimated with training set Prof) fail. The RMSE of the predictions is large compared to the variation of the AGB values and the model fit is poor ($r^2\le 0.33$) in each case.

In Fig. 7 it can be seen that the models severely under-estimate the cumulative probability distribution of AGB for values for AGB greater than 200 Mg/ha in validation set Comm (left sub-figure). This happens regardless of the training set used to train the model, also when the training set is the same as validation set. For validation set Prof, the model over-estimates the cumulative probability distribution of AGB for values of AGB less than 400 Mg/ha regardless of the training set used to train the model (right sub-figure) and for values of AGB greater than 400 Mg/ha the model under-estimates the cumulative probability distribution for each training set.

## Discussion

The results above show that the predictions in study site Gorkha are quite accurate and precise in each case in the cross-validation procedure and no significant difference occurs when different sources of training set data are used. However, there are severe problems in the predictions of AGB in study site Chitwan. The cross-validation procedure shows that there is significant difference in the mean of prediction error when the training set and validation set are different. However, in this study site, the prediction precision overall is weak, the RMSE is large and model fit poor.

In model-based prediction, the correlation between the response, AGB field estimates, and the auxiliary data, LiDAR predictors, define whether the auxiliary data can be used to accurately predict the response over the whole spatial area. In this study, the plot-level prediction map in Fig. 6 and the LiDAR predictor—AGB field estimate correlation in Fig. 3 (right sub-figure) show the basic problem in Chitwan data—the signal (or correlation) between the LiDAR predictors and AGB field estimates is not good enough for precise prediction, and total lack of fit ensues independently of the training set source. Even the predictions of community team plots obtained using community team plots as the training set of the model show no correlation with the field estimates, as seen in the middle left sub-figure in Fig. 6. Even though the error mean is nearly zero, the model fit is poor ($r^2=0.27$). Similar results can be seen for the predictions for professional team plots that are obtained using professional team field measurements as the training set (bottom right sub-figure).

With the lack of LiDAR predictor—AGB field estimate correlation, the predictions in study site Chitwan are dominated by the average values of the field estimates in the training sets. In community team measurements the average of the field estimates is larger than in the professional team measurements. The community plot AGB field estimates are thus heavily under-estimated (22.6 % of the average AGB field estimates) when using the training set of professional team measurements as training set, and overestimated in the opposite case.

It is plausible that the lack of fit of model predictions between different training sets in Chitwan is due to different forest populations used in their sampling. The samples generated separately for each population are not necessarily probabilistic samples on the intersection of the populations, i.e. on closed canopy community forests and bias may ensue. However, the prediction analysis of the various models do not support such an interpretation. In both Gorkha and Chitwan model fit is similar, acceptable in Gorkha and very poor in Chitwan, with all combinations of teaching set and validation set, whether these are cross-validated or self-validated. It seems therefore that the reason to the lack-of-fit in Chitwan is caused by some other effect and not inappropriate sampling.

Model-based prediction of biomass uses field plots for model calibration but they can also be used for model validation. When plot collection is conducted by randomly sampling individual plots or plot clusters, such field campaigns can also be used to test model-based predictions for possible lack of fit.

In the current tests between 50 and 200 plots were measured for two areas of roughly 10,000 hectares each. In heterogeneous forests it is plausible that not every forest type gets an adequate statistical coverage with such a sample. However, even this sampling density far exceeds the cost per area of any foreseeable field sample for nationwide estimates, and is much higher than common sampling densities in national forest inventories. We therefore have to try and use the randomness of sampling as a guarantee against bias at least over sufficiently large forest areas. Once lack of bias has been attained for a model-based estimation method, it becomes much more feasible to provide even high-resolution biomass updates using remote sensing data alone.

## Conclusions

The lessons from this study are positive towards using participatory field measurements. The analyses above show that the LiDAR-data based models calibrated with in situ field estimates conducted by professional foresters or by trained community forest members that use different field sample selection methods, different field sample plot sizes and different methods at the field work itself can be used together without degrading model prediction performance, if the correlation between LiDAR predictors and field estimates is good enough. This was evident in study site Gorkha. In Chitwan, the correlation was poor independent of the source of field measurements leading to imprecise predictions. The combined distributions of AGB field estimates with respect to LiDAR predictors were visually assessed to come from the same distribution in both study sites with both field data, Comm and Prof, see Fig. 3. Thus, there is no evidence that the error in Chitwan predictions is caused by the use of different samples, but from the lack of correlation between LiDAR predictors and AGB field estimates. Thus in both study sites, the prediction results were equally good or bad for both participatory and professional field plot measurements.

Previous studies, e.g. [9], concluded that it is possible to utilize data collected with participatory approaches for traditional forest inventories. The authors of [9] studied the feasibility of participatory REDD+ MRV processes in Tanzania. Their study compared the field estimates from the community forest user groups and professional teams and showed that the mean of AGB field estimates differed by no more than 7 % and mostly by 5 %. The variance was higher in the community measurements, therefore indicating that even though the accuracy was as good as professional measurements the precision was weaker.

These types of participatory inventories are limited in the geographic representativeness [10], especially when the aim is the estimation of carbon resources for the remuneration of local communities, since the Community Forests are relatively small compared to the landscape scale.

The present study is the first attempt of its kind to utilize field data collected by community people in LiDAR-based biomass inventories within the REDD+ context. The implementation of participatory methods for the monitoring & measuring, reporting and verification (M&MRV) of forest carbon credits with high accuracy and resolution is a fundamental step for the implementation of REDD+ projects in community forestry level.

The results of this study support the conclusions of a side event on evolving requirements and solutions for REDD+ monitoring, with community focus, at the UN climate change conference in Warsaw in 2013 (COP19) [11]. The side event concluded with the agreement that community monitored data can be scientifically accurate and support also new technology, such as LiDAR. But concerns were raised on whether community monitoring of carbon performance can form the basis for broader financial rewards for REDD+ and whether the data can be integrated into a broader national forest monitoring systems.

This study agrees with the study by [12]. They reported that the overall aboveground biomass estimated by community members differed only slightly from the estimates by the professional foresters. The results of this study therefore show that it is possible to use calibration plots measured by community people in model-based predictions of above-ground biomass. They also show that a model-based analysis can be used to validate the accuracy of field plots by calculating predictions with a model based on different subsets of the plots. If the model predictions thus obtained are compatible and consistent, the field estimates can be regarded as reliable. This approach gives ownership of verified data to different stakeholders which is key to implementing performance based financing mechanism.

The approach described here will hopefully be helpful for unbiased monitoring, reporting, and verification under a result-based payment mechanism in which plot data collected by local communities are integrated with advanced remote sensing-based measurements.

## Methods

### Study area

The study area consists of two separate sites, Kayarkhola watershed in Chitwan (labeled as Ch) and Ludikhola watershed in Gorkha (Go) located in Nepal, see Fig. 1 for the site locations. The sites are located quite near each other, the distance from northernmost part of Chitwan area is about 20 km to the southernmost part of Gorkha area. The study area in Chitwan is about 12.2 km from west to east, 8.0 km from south to north. The distances in Gorkha are about 10.6 km from east to west, 6.5 km from south to north.

The Kayarkhola watershed is located in Chitwan district, which is a part of the Central Development Region of Nepal. Its total area is 8,002 hectares (ha) and it consists of tropical to sub-tropical forests, covering an altitudinal range of 245–1944 m [5] with 16 Community Forest User Groups (CFUGs) managing this forest. The watershed consists of three different types of forest namely Sal forest, mixed hardwood forest and Riverine forest [13]. The watershed is inhabited by socially and ethnically diverse forest-dependent indigenous communities such as the Chepang and Tamang [14]. These ethnic groups are some of the most marginalized ethnic groups in the country. Chepang and Tamang communities practice shifting cultivation which puts severe pressure on forest resources. The REDD+ pilot project implemented in the area plays a major role to address the issues of forest degradation and deforestation by promoting sustainable forest management practices and linking it with the REDD+ incentive mechanism [13].

The Ludikhola watershed in Gorkha district is located in the southern part of Gorkha. The watershed is located in the Hill region characterized by sub-tropical broad leaved forests, ranging from 318 to 1714 m above sea level. The total area of the watershed is 5750 ha, out of which 4869 ha is forest area, 632 ha is agricultural land and the rest is barren, grassland and natural water bodies. There are 31 CFUGs managing an area of 1888 ha of forests as Community Forests (CFs). The forest in Gorkha represents sub-tropical forests. The watershed was heavily deforested in the past and this has been controlled through sustainable community forest management and conservation through REDD+.

Dominant forest types in the study area are hill sal (*Shorea robusta*) forest, and Schima-Castanopsis forest. Even though Shorea robusta mixed subtropical hill deciduous forest forms the major forest type in Kayarkhola (Chitwan) and Ludikhola (Gorkha), associated species varies between these two watersheds. *Lagerestroemia parviflora*, *Mallatus phillipinensi* and *Terminelia tomentosa* are dominant associates in Kayarkhola (Chitwan) whereas *Schima wallichii* and *Castanopsis indica* are the most common associates in Ludikhola (Gorkha). According to broader climatalogical categorization of forests, forests in Kayarkhola fall under tropical broadleaved forests and in Ludikhola the forests are of sub-tropical broadleaved forest mostly, with *Shorea robusta* and *Schima wallichi* (sal and chilaune) as principal dominant species.

### LiDAR data

Airborne discrete-return LiDAR data was acquired in February/March 2011 from the two watersheds. The two watersheds were scanned in full coverage from 2200 m average height above ground using a local helicopter equipped with a Leica ALS50-II lidar-scanner device. The helicopter flight path was east-west strips at 1 km distance. The scanning parameters are presented in Table 3. The collected LiDAR data were evaluated after each flight, and supporting scans were conducted if data gaps or other problems occurred.

**Table 3:** Specifications for the LiDAR scanning data

Parameter	Value
Average flying altitude above ground level	2200 m
Flying speed	80 knots
Sensor pulse rate	52.9 khz
Sensor scan speed	20.4 lines per second
Nominal outgoing pulse density at ground level	0.8 points per square meter
Scanning field of view (FOW) half angle	20°
Swath width at ground level	1601.47 m
Point spacing on the ground (across-track / along-track)	max. 1.88/2.02 m
Geometric accuracy (horizontal and vertical)	max. 1 m

Raw LiDAR data were classified by the vendor into three categories: ground, vegetation and error returns. Further pre-processing included calculation of a digital terrain model (DTM) from the ground returns, removal of overlaps from the raw data, and conversion of height coordinates (zvalues) of the vegetation returns from absolute elevation into distance-to-ground using the DTM [15]. Overlap removal is a procedure to ensure uniform density of points for estimation by the area-based approach (ABA). ABA methods use quantized vertical histograms as the regression variables and it is seen as desirable that their sampling noise, i.e. density of LiDAR points per square meter, is uniformly distributed. From the pre-processed LiDAR data, several LiDAR features were estimated in order to serve as the LiDAR-predictors in the AGB prediction model. The features have been taken from [16] and are an extended and modified version of those published by [17]. They include: (1) different height percentiles for the first-pulse and last-pulse returns, (2) mean height of first-pulse returns above 5 m (high-vegetation returns), (3) standard deviation for first-pulse returns, (4) ratio between first-pulse returns from below 1 m and all first-pulse returns, and (5) ratio between last-pulse returns from below 1 m and all last-pulse returns. These features were estimated from LiDAR points within the plot footprints described below.

### Field samples

Field sample plots were selected with two different methods, depending on the inventory team. The professional forest measurement teams and forest measurement teams coming from the local communities collected the field data during the year 2011. The selection criteria for the field plots were different among the two groups of forest measurement teams.

Field plot center coordinates were recorded using Differential GPS (DGPS) with ProMark 3 and MobileMapper CX devices, and corrected in post-processing mode (GNSS Solutions software and MobileMapper Office software). Plots were located with a family of GPS devices where one device is left stationary for all day and it provides differential correction to all other GPS devices used in positioning the plots within a cluster of plots. Subsequent off-line DGPS post-processing was also used and plot geo-location error was estimated to be less than 1 m.

The professionally collected plots were collected as a part of a much larger campaign addressing the REDD+ program in Nepal. This larger program requires sampling from a national, officially accepted forest mask. Since that nationally accepted forest mask does not admit auxiliary information, such as vegetation index, elevation or aspect, clustered random sampling with uniform probability on the area of interest covered with LiDAR was used.

#### Sampling design and field plot design used by community teams

A stratification was done where forests with more than 70 % of canopy cover were considered as dense strata (i.e. closed canopy) and less than 70 % as sparse strata (i.e. open canopy). In a post-processing step the classification between open and closed canopy was revisited based on LiDAR pulse density so as to obtain a uniform classification of closed canopy for both sets of field plots. Plots deemed not to fall on closed canopy forest were eliminated from the sample by a 70 % canopy cover criterion. The 70 % canopy cover was used as a surrogate variable for selecting as similar a sample of community plots as possible to the professional plot exclusion policy. This 70 % canopy cover was computed as the proportion of vegetation points of all LiDAR points. Since plot sampling was clustered or simple random sampling for professional and community plots, respectively, it was assumed that the plots satisfying the canopy cover criterion reflect different AGB classes in their respective statistical proportions and the estimates are therefore unbiased.

Forest stratification was carried out using high resolution remote sensing imagery (GeoEye image) in ERDAS and ArcGIS software. The random permanent plots that were established during baseline survey were measured for the purpose of monitoring. A total of 365 permanent plots were measured in the field. There were 298 plots in closed canopy forests and 67 plots in open canopy forests.

The size of the plot was fixed to a circle of 8.92 m radius. A sub-plot of 5.64 m radius was established for saplings and a sub-plot with 1 m radius was established for counting regeneration.

#### Sampling design and field plot design used by professional teams

Before the field campaign, the location of sample plots was designed using a systematic clustered random sampling method. Each cluster contained eight sample plots. Within the clusters, the sample plots were aligned in two parallel columns in North-South direction, with four plots per column. The distance between plots was 300 m, both between columns and rows. The original sampling design generated 16 clusters for the Kayarkhola watershed with a total number of 112 plots while it included 15 clusters for a total of 115 plots for the Ludikhola watershed. The actual number of plots available for the purpose of the study is less than that because some plots were either placed outside the area of study or in non forested areas (water, agricultural and bare soil areas). The total number of plots available for the study was therefore 57 for Kayarkhola and 92 for Ludikhola. The plots were of fixed circular shape with a 12.62 m radius, equivalent to an area of 500 m$^2$.

The field data were collected in April/May 2011. All the sample plots that were located in forest with at least 10 % canopy cover were measured in the field. The measurements at tree-level included all living trees and shrubs above 5 cm diameter within the plot area.

#### Above ground biomass measurement estimates

Within each plot, individual tree diameter at breast height measurements for both live and dead trees were taken and used in allometric equations given in [18] to estimate above ground biomass, AGB (stem, branch and foliage biomass). The individual tree AGB field estimates were totaled for each subplot and converted to AGB Mg/ha.

Large AGB field estimate values (over 1500 Mg/ha) are assumed to be outliers, caused by e.g., plot-level AGB estimation errors or measurement errors. At plot level, with relatively small plots, it sometimes occurs that one or a few very thick trees cause the polynomial formula for plot level volume computation to become unstable because of extrapolation. This extrapolation error may cause the volume of a single exceptional tree to be estimated so high that a timber volume of more than 2000 m$^3$ per hectare is attained, which is not realistic. There is no adequate statistical data available to quantify this phenomenon and revisiting the plots for validation is not feasible either. We therefore resorted to manual removal of probable outliers that are detected as statistical outliers of AGB field estimate distribution and also by visual interpretation from LiDAR predictor—AGB field estimate scatterograms.

In field measurements of the local teams, there were two plots in which the estimated AGB was very high, 2202.6 Mg/ha and 3257.6 Mg/ha, respectively, and could be assumed as outliers. Visual assessment of the LiDAR predictor—AGB field estimate distribution supported this decision. Thus these two plots were discarded as outliers. Otherwise, the estimated AGB values of the plots are treated as the ground truth. After deletion of outliers, a total of 372 field plots measured by local forest measurement teams (182 in the study site Chitwan, 190 in the study site Gorkha), and a total of 149 field plots by the professional measurement teams (57 in Chitwan, 92 in Gorkha) were available for this analysis.

**Fig. 8:**
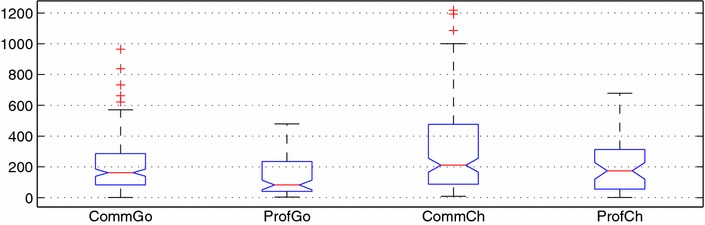
Boxplots of the AGB field estimates

Figure 8 shows the variability of AGB field estimates in each dataset without outliers. Especially data collected by the local forest inventory teams in the study site Chitwan (CommCh) contain a significantly larger range of values than the other subsets. Also, the averages of the ABG field estimates in the field plots measured by local measurement teams were larger than those estimates by the professional inventory teams in both study sites, Gorkha and Chitwan.

### Differences between field estimates due to different sampling designs

Different sampling designs affect the distribution of AGB field estimates due to different characteristics of the forest in which the sample plots are located. In particular, the ownership of the forest affects the distribution average and median values, see Table 4 and Fig. 2. The sample collected by local community teams contain fewer plots from privately owned forests compared to samples collected by professional teams. After discarding those plots from the sample of local community teams, i.e., considering only plots within community owned forests, the average and median values of AGB are larger compared to AGB values obtained by considering all the plots in the professional teams’ sample. This effect does not happen in the sample of local community teams. Also, in the case where only plots within closed canopy forests are considered, the average AGB values are slightly larger than in the case where all the measured plots are included.

**Table 4:** Sizes and average AGB values (Mg/ha) of different measurement team dependent subsets in Chitwan and Gorkha areas

Subset	Comm		Prof	
	*N*	$\overline{\mathrm {AGB}}$	*N*	$\overline{\mathrm {AGB}}$
Gorkha				
All	190	203.3	92	127.3
Community owned	184	202.9	45	190.4
Closed canopy	157	216.5	82	133.5
Community owned and closed canopy	151	216.5	41	198.3
Chitwan				
All	182	308.4	57	205.8
Community owned	178	313.8	31	290.3
Closed canopy	154	318.8	48	207.0
Community owned and closed canopy	151	323.7	26	298.0

When only the plots in dense community owned forests are considered, the average AGB values obtained are the largest. The sizes of these subsets are relatively small (only 26–41 field plots). This small sample size may cause problems in the model-based prediction of AGB.

### Validation procedure

In this study, a method based on a linear model to predict the AGB values, namely a Bayesian linear model with orthogonal predictors resulting from truncated singular value decomposition is used [19]. This model is designed to give accurate and precise predictions when using a small training set size compared to the number of possibly correlated predictors. It utilizes the singular value decomposition of the normalized predictors, and allows bigger deviation from zero to the regression parameters of the orthonormalized predictors which are known to explain the original predictor variability most, i.e., have biggest singular values. With this method, the effective number of predictors is cut down according to the given data and predictor explanation ratio, and thus it performs better especially with small training sets.

The characteristics and distributions of the AGB field estimates vary among the subsets, e.g., subset CommCh contains data with highest values of AGB, subset ProfGo the lowest values. Variation can be seen also in the LiDAR predictor values among different subsets. If the data can be considered as samples from the same distribution, i.e., there are no significant differences in the forest characteristics nor in the field sample measurement routines, a common model based on all these data should perform well. That is, the prediction model where model parameters are estimated with all the data (in this study Comm + Prof), or with some subset of data (Comm or Prof), should give equally accurate estimates, both in the data belonging to the teaching set subset, or to the other subsets [20]. Thus, to validate whether the data come from the same distribution and if data from one subset can be used to predict the AGB field estimates of another, cross-validation procedure is used. Each subset (Comm and Prof) at time serve as the validation set and the predictions estimated with different subsets (Comm, Prof and Comm + Prof) are validated.

To verify the prediction performance of different data validation subset of size $N_v$ on different models, root mean square error,

1 $$\begin{aligned} \mathrm {RMSE} = \sqrt{\frac{\sum _{i=1}^{N_v}\left( \tilde{y}_{v,i}-y_{v,i}\right) ^2}{N_v}}, \end{aligned}$$

mean difference,

2 $$\begin{aligned} \mathrm {D}\, = \frac{\sum _{i=1}^{N_v}\left( \tilde{y}_{v,i}-y_{v,i}\right) }{N_v}, \end{aligned}$$

relative RMSE ($\mathrm {RMSE}\,\% = \mathrm {RMSE} \times 100\,\%\,/\overline{y}_v$) and relative mean difference ($\mathrm {D}\,\% = \mathrm {D} \times 100\,\%\,/\overline{y}_v$) are used. Here $\tilde{y}_{v,i}$ is the the predicted AGB value for plot *i*, $y_{v,i}$ is the corresponding field estimate (“truth”), and $\overline{y}_v=\sum _{i=1}^{N_v} y_{v,i}/N_v$.

The error statistics are calculated using both leave-one-out cross-validation (LOOCV) procedure and straight cross-validation, depending on the case. In a case, where the training set contains the same plots as the validation set, for example in the full dataset case (Comm + Prof) and self-validation cases (training and validation sets are the same), LOOCV is used. In LOOCV, one plot of the training set, *i*, at a time is used as the validation plot, and the rest of the training set is used to estimate the model parameters which are then used to predict the validation plot AGB. With the predicted values $\tilde{y}_{v,i},\ i=1,2,\ldots ,N_v$ the error statistics are calculated using formulas (1) and (2). In case where the AGB field estimates of one subset are predicted using a model calibrated with another subset (e.g. validation set Comm plots are predicted using training set Prof), the calibrated model is used as such to predict all the values of the other subset and the error statistics are calculated in a straightforward manner using the given formulas.
